# Identifying barriers to the use of ultrasound in the perioperative period: a survey of southwestern Ontario anesthesiologists

**DOI:** 10.1186/s12913-019-4040-2

**Published:** 2019-04-04

**Authors:** J. Chui, R. Lavi, A. F. Hegazy, P. M. Jones, R. Arellano, H. Yang, D. Bainbridge

**Affiliations:** 1 Department of Anesthesia & Perioperative Medicine, Schulich School of Medicine and Dentistry, Western University, London, ON Canada; 2 Department of Epidemiology & Biostatistics, Schulich School of Medicine and Dentistry, Western University, London, Canada

**Keywords:** Ultrasound, Point of care ultrasound, Anesthesia, POCUS, Ultrasound guidance, Central venous catheter

## Abstract

**Background:**

Ultrasound (US) can be used for many perioperative procedures, but evidence is lacking as to its frequency of use and barrier of application. The objectives of this survey were to determine i) how often US guidance was used perioperatively for vascular access placement, nerve blocks, and heart and lung assessment, and ii) to identify the barriers and the limitations of using US amongst anesthesiologists in southwestern Ontario.

**Methods:**

We conducted a web-based survey in over 40 academic or community hospitals at southwestern Ontario.

**Results:**

Of 266 surveys sent, 66 complete surveys were obtained (response rate of 25%). Most respondents (> 80%) reported that US was commonly used for central venous catheter (CVC) insertion, followed by regional blocks; the uses were less frequent for neuraxial blockade and cardiopulmonary assessment. Most respondents wanted to use US more frequently as part of their practice and felt that they already had adequate US training. However, most respondents (59%) reported limited access to US machines in their working institutes as being the major barrier to incorporating US in their daily practice.

**Conclusion:**

The most common uses of US in anesthesia practice in southwestern Ontario were for CVC insertion and regional blocks. Most anesthesiologists in southwestern Ontario are interested to incorporate US in their daily practice but most were limited by the lack of US resources. Apparently, only providing knowledge and skills teaching may not be sufficient to further improve the US utilization in our region; a matched administrative effort appears to be the next challenge.

## Background

Ultrasound (US) guidance plays an important role in Anesthesia practices to improve patient safety and procedural efficacy, and to aid patient assessment. The uses of US guidance in anesthesia have been shown to reduce the failure and complication rates and the number of cannulation attempts during central venous catheter (CVC) insertion and regional blocks [1–3]. Multiple national clinical practice guidelines, including those issued by the National Institute for Health and Care Excellence (NICE) [4] in the UK, and the Canadian Anesthesiologists’ Society [5], have recommended the routine use of US guidance when performing invasive procedures and that dedicated US capability must be provided. Despite these recommendations, the routine use of US guidance has not been widely adopted by anesthesiologists in Ontario [6]. A previous survey [6] of anesthetic practice in Ontario at 2011 found a slow adoption of US for CVC insertion and regional nerve blocks, and identified lack of training and perceived need as the major barriers of US utilization, indicating a knowledge gap and deficit of training opportunities in our region.

With more recent technological advances and an apparent increasing popularity of US in anesthesia, the uses of US have been expanded to include guiding more technically demanding anesthetic procedures such as arterial cannulation and neuraxial blockade. At the same time, the interest of using Point-Of-Care US (POCUS) to provide bedside diagnostic cardiopulmonary functional imaging has been rapidly growing. Because of the rapid evolution of the evidences and the widened application of US in anesthesia, we hypothesized that there were temporal changes in US practices in southwestern Ontario, affected by changing barriers and limitation to US utilization, as well as evolving teaching preference in both content and approach to learning US applications. We also suspected there would be differences because of a different target population from the previous surveys [7–15]. As such, we conducted a survey to determine how often US guidance is used perioperatively for different applications and identify the barriers and the limitations of using US guidance (both on a personal and institutional level), assess the general attitude towards the use of US guidance, and identify interest in perioperative US training and the preferred methods of education amongst the anesthesiologists in southwestern Ontario.

## Methods

After local ethics approval (REB#108969; Apr 2017 approved), we conducted a web-based survey to assess the current perioperative practice of using US for vascular access placement, nerve blocks and heart and lung assessment among southwestern Ontario anesthesiologists in over 40 healthcare centers (Appendix 1). An electronic signed written consent was obtained from all participants.

### Participants

This survey was targeted to practicing anesthesiologists in southwestern Ontario working at either an academic, community or private healthcare centers. The geographic boundary of southwestern Ontario is bounded by Lake Huron to the north; the St. Clair River to the west; and Lake Erie to the south; Central Ontario and the Golden Horseshoe to the east. The region had a population of 2.5 million in 2016.^1^ We targeted only anesthesiologist working in southwestern Ontario because this was the only feasible sample we could obtain a complete contact of the all potential respondents to avoid sampling errors. We did not survey through the professional society/organization membership email list across Canada and Ontario because their membership email lists only represent a fraction of working anesthesiologists in Canada or Ontario (see Discussion). A 20–40% response rate was expected based on the response rate of the previous electronic survey in Ontario [6].

### Survey development

The survey was first constructed by the investigators, and then modified with the recommendations from a survey methodology expert at Western University, Ontario. We further validated the survey by pilot testing on 5 anesthesiologists and 5 anesthesia residents/fellows to ensure appropriate sentence construction and sufficient data collection. The survey was hosted on Qualtrics survey™ (https://qsharingeu.eu.qualtrics.com). The online administration of the survey in Qualtrics was tested in different browsers and platforms to ensure smooth conduct.

The survey was composed of 48 questions and was estimated to take approximately 30–45 min to complete. The questions were designed to address five areas: i) participant characteristics, ii) institution characteristics, iii) participants’ practice, iv) barriers of using US (e.g. anesthesiologists’ perceived need, logistics and institutional policy), v) training preferences (e.g. preferred learning method).

### Survey administration

Contact information for all Site Chiefs of Anesthesia Departments in southwestern Ontario was obtained from our administrator. We manually telephoned all department executives or site chiefs to introduce the study and requested local promotion of the study. We were successfully able to establish a connection to the executives of 196 anesthesiologists working in 28 institutions in southwestern Ontario, who agreed to both distribute the survey on the teams behalf and promote the study locally. We were further able to contact the local executives of additional 70 anesthesiologists in 7 institutes, who agreed to forward the survey to the working anesthesiologists in their Departments but refused to promote the study locally on behalf of the study team. We sent the survey directly to these 70 anesthesiologists. There were no email addresses or contact information available for 36 anesthesiologists. Thus, we were able to deliver the survey invitation to 266 practicing specialists out of a total of 302 anesthesiologists in our region.

The survey was distributed by e-mail in January 2018, with a subsequent reminder e-mail sent every 2 months thereafter for a total of 3 reminders. To optimize the response rate, we provided an incentive (a draw for an iPad Mini) for completing the survey.

### Statistical analysis

The survey data were stored on the Qualitrics™ host server and downloaded for analysis following completion of data collection. The data were analyzed using Stata® [version 14.0] (StataCorp LLP, College Station, Texas). Descriptive statistics, including mean, standard deviation (SD), frequencies, and percentages, were reported as appropriate to assess the qualitative aspects of the data. No inferential statistical analysis was performed.

## Results

The survey was distributed to 266 anesthesiologists who were currently working in southwestern Ontario. Sixty-six complete surveys were obtained yielding a response rate of 25%. The demographics of respondents and their practising institutes are summarized in Tables 1 and 2.

**Table 1:** Participant Characteristics

	N (%)
Female sex^a^	17 (26)
Age group	
• 30–39	27 (41)
• 40–49	12 (18)
• 50–59	19 (29)
• 60–69	6 (9)
• > 60	1 (2)
• Prefer not to answer	1 (2)
Consultant Anesthesiologist^a^	55 (85)
GP/FM Anesthesiologists	8 (12)
Practice at one hospital	23 (35)
Practice at more than one hospital	43 (65)
Community Hospital	33 (50)
Academic Teaching Hospital	31 (47)
Private Hospital	1 (2)
Size of the institute	
• Large	32 (49)
• Medium	26 (39)
• Small	8 (12)
Year of Practice	
• Less than 5 years	22 (33)
• 5–10 years	9 (14)
• 11–15 years	8 (12)
• 16–20 years	6 (9)
• Over 20 years	21 (32)
Full time (4–5 days/week)	60 (91)
Part time (2–3 days/week)	6 (9)
Canadian medical graduate	43 (65)
International medical graduate	23 (35)
Training qualification	
• Completed fellowship in Anesthesia	33 (29)
• Master’s degree	10 (9)
• PhD degree	3 (3)

^a^2 respondents did not answer this question

**Table 2:** Institution characteristics

	N (%)
No of operating rooms in your institute	
• 1–3	8 (12)
• 4–9	21 (32)
• 10–15	9 (14
• 16–20	20 (30)
• > 20	8 (12)
No of ultrasound machines are designated for perioperative care at your institute	
• 0	6 (9)
• 1	18 (27)
• 2	16 (24)
• 3	11 (17)
• 4	8 (12)
• ≥5	3 (5)
• Not sure	4 (6)
The ultrasound machines are readily available for use whenever I may need them.	
• Yes	37 (57)
• Sometimes	19 (29)
• No	9 (14)
The use of ultrasound at my institution is common practice for central line insertion:	
• Yes	54 (82)
• No	5 (8)
• Don’t know	7 (11)
The use of ultrasound at my institution is common practice for regional blocks:	
• Yes	53 (80)
• No	11 (17)
• Don’t know	2 (3)
The use of ultrasound at my institution is common practice for difficult arterial line placement:	
• Yes	41 (62)
• No	19 (29)
• Don’t know	6 (9)

The majority (74%) of the respondents were male; 41% were 30–39, 18% were 40–49, and 29% were 50–59 years of age. The vast majority (85%) of the respondents were consultant anesthesiologists. Only a small minority (12%) were general practitioner/family physician anesthesiologists. Half of the respondents worked in a community hospital setting and the other half worked in academic teaching institutes. The most common size of the institutes was either medium (10–20 operating rooms running daily) (39%) or large (> 20 operating rooms running daily) (49%). The vast majority of respondents (91%) were full time anesthesiologists. Most (65%) practised either < 5 years or > 20 years;

In terms of institution characteristics, most (> 80%) reported the use of US was common practice for CVC insertion and regional blocks at their institutes. The use of US for arterial cannulation was a common practice for more than half of respondents’ institutes. However, the vast majority of respondents reported limited access to US machines; 9% had no US machine, 27% had 1 machine, 24% had 2 machines, and 17% had 3 machines (Table 2). There was an average of 6.2 (SD 3.3) operating rooms per US machine. Nine percent of respondents did not have any US machine available in their institutes. Only half of the respondents could have readily access for US machine whenever they were required.

### US practices

A vast majority of respondents (89%) reported “always” or “frequent” uses of US for CVC insertion. The use of US for arterial cannulation was quite variable and was less common than for CVC insertion (Table 3).

**Table 3:** Participants’ practice

	Always	Frequently	Sometimes	Seldom	Never	As clinically indicated
I use ultrasound guidance when inserting a central line	54 (82)	5 (8)	4 (6)	1 (2)	1 (2)	1 (2)
I use ultrasound guidance for arterial cannulation	3 (6)	16 (24)	23 (35)	13 (20)	7 (11)	4 (6)
I use ultrasound guidance when performing a regional block	36 (57)	6 (10)	7 (11)	1 (2)	11 (18)	2 (3)
I use ultrasound guidance for spinal and epidural blocks placement	0	1 (2)	6 (9)	17 (27)	39 (61)	1 (2)
I use ultrasound to assess the heart and lung whenever indicated	6 (9)	7 (11)	12 (18)	14 (21)	21 (32)	6 (9)

All results in the table were present in N (%)

Despite the well-established benefit of US-guided regional anesthesia techniques, almost half of the respondents did not routinely use US to perform regional blocks. A small minority of the respondents (18%) reported they never use US for regional nerve blocks. The vast majority (85%) of the respondents never or seldom used US for neuraxial anesthesia. No respondent used US routinely for neuraxial blocks. Half of the respondents never or seldom used US for the assessment of cardiac and pulmonary abnormalities.

### Barrier of using US in practice

The majority (68%) of the respondents agreed that US was used on a regular basis at their institution. The majority of the respondents wanted to use ultrasound guidance more frequently as part of their perioperative care and felt that they already have adequate training to use US regularly. None agreed that they do not need to use ultrasound guidance in their practice. However, more than half (59%) of the respondents reported inadequate access to the US machines on a regular basis (Table 4). The majority (58%) of the institutes did not have an existing policy on the use of US guidance; 29% of respondents were not sure whether there was an existing policy in their working institutes. Only a small proportion (21%) of respondents felt they were always encouraged to use US by the clinical leadership at their institutions.

**Table 4:** Barriers of using Ultrasound

	Always	Frequently	Sometimes	Seldom	Never	As clinically indicated
I want to use ultrasound guidance more frequently as part of my perioperative care:	26 (39)	20 (30)	15 (23)	3 (5)	2 (3)	0 (0)
I feel like I have adequate training to use ultrasound regularly:	17 (26)	17 (26)	20 (30)	9 (14)	2 (3)	1 (2)
I do NOT need to use ultrasound guidance in my practice:	0	0	2 (3)	9 (14)	15 (23)	40 (61)
The institution where I practice does NOT have enough ultrasound machines for me to use on a regular basis:	14 (21)	9 (14)	16 (24)	7 (11)	11 (17)	9 (14)
Ultrasound is used on a regular basis at the institution where I practice:	22 (33)	23 (35)	9 (14)	7 (11)	4 (6)	1 (2)
I am encouraged to use ultrasound by the clinical leadership at my institution:	14 (21)	15 (23)	20 (30)	3 (5)	7 (11)	7 (11)

All results in the table were present in N (%)

Some respondents have provided written (free text) feedback in the survey expressing the lack of resources was the major hindrance of US applications in their practise. They were frequently discouraged by the lack of availability of US equipment (e.g. borrowing US machine/probe from the intensive care unit). This general lack of resources also resulted in a lack of momentum within the local anesthesia group to incorporate US into their practice.

### Training preference

All respondents reported they have received some forms of US training in the past. This included during their residency or fellowship training, peer-to-peer learning, participating in single/multiple day workshops, reading textbooks or electronic journals (Table 5). The vast majority of the respondents agreed that US guidance is an important skill for all anesthesiologists and they could see the benefit of US training. Most respondents (72%) would like to receive additional ultrasound training; however only half reported they had planned to acquire additional training in the coming 12 months. All respondents agreed that they would be benefit from training via E-learning modules. For those who did not plan to acquire more training in the coming 12 months, most reported they had sufficient training and some reported there was not enough time or resources available.

**Table 5:** Training Preference

	N (%)
I have received the following training in ultrasound use:	
• None	0 (0)
• Residency training	33 (50)
• Fellowship training	20 (30)
• Peer-to-peer	38 (58)
• Single day workshop	36 (55)
• Multiple day workshop	32 (49)
• Electronic format- internet based	36 (55)
• Printed material (textbook, brochures, pamphlets, etc)	36 (55)
I would like to receive ultrasound training:	
• Yes	47 (72)
• No	18 (28)
I would benefit from participating in ultrasound training via E-learning modules (only to “yes” on above question):	
• Strongly Agree	12 (40)
• Agree	9 (30)
• Somewhat Agree	9 (30)
• Somewhat Disagree	0 (0)
• Disagree	0 (0)
• Strongly Disagree	0 (0)
I plan to undertake ultrasound training (additional or for the first time) in the next 12 months:	
• Yes	30 (46)
• No	36 (55)
I am NOT planning to undertake training in the use of ultrasound because:	
• I have sufficient training	14 (21)
• There is no time or resources available for training	10 (15)
• It is not needed for my practice	3 (5)
• I have no interest in ultrasound training	0 (0)
• Not answered	29 (44)
I do NOT see the benefit to ultrasound training:	
• Strongly Agree	0
• Agree	0
• Somewhat Agree	0
• Somewhat Disagree	9 (14)
• Disagree	17 (26)
• Strongly Disagree	40 (61)
Ultrasound guidance is an important skill for all anesthesiologists to have:	
• Strongly Agree	50 (76)
• Agree	10 (15)
• Somewhat Agree	5 (8)
• Somewhat Disagree	1 (2)
• Disagree	0 (0)
• Strongly Disagree	0 (0)

## Discussion

We performed a regional survey representing the use of US in the perioperative period in a small geographic region in southwestern Ontario. In our locality, the use of US in anesthesia was a very common practice in both academic and community institutes. Most respondents used US for procedural guidance; mainly for CVC insertion, and it was less commonly used in regional nerve blocks and arterial cannulation. The use of US for cardiopulmonary assessment for diagnostic purposes was infrequent. Ultrasound was least commonly used for neuraxial blockade.

The key barriers identified for using US in the perioperative period was the lack of US machines, an average of 6.3 operating rooms per machine. Some respondents expressed the frustration of limited US resources in achieving their desired safety standards. Most respondents reported they had received adequate US training and wanted to incorporate US in their practices. This differs from previous surveys which suggested that inadequate training or lack of motivation were the main barriers to utilization [6].

### Interpretation

The current practice pattern of the US utilization in anesthesia in southwestern Ontario was not entirely surprising; US was more frequently used for CVC insertion and regional nerve block than either arterial cannulation or cardiopulmonary assessment. A previous survey [6] of 203 anesthesiologists conducted in Ontario in 2011, found approximately 30–40% of the anesthesiologists used US routinely for CVC insertion and regional anesthesia. Our survey showed an increase in the adoption of US since the last survey [6]. The NICE guidelines state that “US guidance should be used in most clinical circumstances where CVC insertion is necessary and that all those involved in placing CVCs using US guidance should undertake appropriate training to achieve competence.” [4] Similarly, the use of US in peripheral nerve block have been shown to reduce block performance time, increase block success, improve block quality and allow adequate visualization of surrounding structures, needle and catheter (ASRA guideline: Level Ib evidence) [16]. These benefit have resulted in better patient safety by reducing local anesthetic systemic toxicity (ASRA guideline: Level Ia evidence) and reducing hemi-diaphragmatic paresis (ASRA guideline: Level Ib evidence) [16]. Our current practice pattern appears discordant with the current recommendation.

The adoption rates of US for arterial cannulation, neuraxial blockade and cardiopulmonary assessment were low, which might be related to these being relatively new techniques in Anesthesia, have fewer demonstrated benefits as well as limited recommendations from clinical guidelines. Although US-guided arterial cannulation has been shown to improve first-pass success and reduce the number of attempts [17, 18], the current guidelines [19, 20] do not recommend routine real-time US. Similarly, the use of US in neuraxial blockade shortens the procedural time, improves block success and allows better prediction of epidural depth (ASRA guideline: Level 1b evidence). The current guidelines do not strongly advocate the routine use of US. Point-Of-Care assessment of the heart, lungs and volume status with US has become more frequent among Anesthesiologists. Early evidence suggests that POCUS is associated lower mortality [21] and modifies anesthetic technique in hip fracture surgery [22], and frequently alters management in patients with suspected cardiac disease in preoperative clinic [23].

One major finding of this survey was the lack of US resources is a key barrier of using US in the perioperative period. Contrary to the Canadian Anesthesiologist Society (CAS) practice guideline [5] which stated states “For the placement of central venous catheters, dedicated ultrasound capability must be provided.”, this general lack of US resource that impedes the safe conduct of anesthetic procedures should call attention to the relevant administrative bodies for appropriate resources allocation. However, the lack of US resources does not only occur within the subspecialty of Anesthesia in Ontario. A survey [24] of rural Ontario physicians reported that approximately 40% respondents did not have an ultrasound machine available in their emergency department. Ironically, the uses of US guided CVC insertion [25] and regional nerve block [26] were found to be cost-saving compared with landmark technique, corresponding to saving £ 2000 per 1000 CVC insertion [25] and $47.3 per regional nerve block [26]. Given the rapidly diminishing cost of small hand held ultrasound machines economics should not be a factor in the acquisition of ultrasound devices.

### Strengths and limitations

Our study was limited by the low response rate but comparable to the response rates in other published electronic surveys in the Canadian anesthesia population [6]. We have attempted to use a more focused survey in a small geographic region with efforts to make personal contact, to improve the response rate; however, this had limited success. As with other surveys, our results suffer from non-responder bias, the non-responders of the survey were likely the one who had less interest in US.

One possible strength of this study was the known denominator of all working anesthesiologists in southwestern Ontario for this survey. Compared to the methodology of the previous survey that was conducted based on the professional society available contact information, the sample might not truly represent the target population. We did not survey through the CAS or Ontario Medical Association membership email list across Canada and Ontario because their membership email lists only represent a fraction of working anesthesiologists in Canada or Ontario. For instance, there were approximately 2600 members of Canadian Anesthesiology Society at 2018 but there was 3744 Anesthesia Specialist registration at the Royal College of Canada Directory in the same year. We did not use the email list on the College of Physicians and Surgeons of Ontario because many community anesthesiologists did not list their personal email contact information in the public domain, resulting in a potential biased and incomplete sample. Arguably, the results of our survey may not necessarily be generalizable to the all other geographic regions or the whole country; however, it may provide a reasonable insight to the regions with similar resources and settings.

## Conclusion

In conclusion, the uses of US in Anesthesia at Southwestern Ontario were common, but the uses were less frequent in the relatively new application such as neuraxial blockade and POCUS assessment. It appears that the anesthesiologists in Southwestern Ontario have a great interests and motivation to use US in their practice, but most were unable to achieve a true day-to-day incorporation because of the limitation of US resources. Apparently, at this stage, 2 decades since US was introduced in Anesthesia Practice, only providing knowledge and skills teaching is not sufficient to further improve the adoption of the US in our region; a matched administrative effort with adequate resource allocation appears to be our next challenge in our journey to incorporate US in our daily practice.

## Appendix

### Participants institutions in Southwestern Ontario

St. Thomas Elgin General Hospital

Stratford General Hospital

St. Mary’s General Hospital (St. Mary’s)

Clinton Public Hospital

Windsor Regional Hospital Ouellette Campus

Windsor Regional Hospital Met Campus

Hotel-Dieu Grace Hospital Windsor

Chatham Kent Health Alliance

St. Joseph’s Health Sciences Association of Chatham, Inc.

Leamington District Memorial Hospital

Bluewater Health (Sarnia General Hospital)

Charlotte Eleanor Englehart Hospital (Sarnia Bluewater)

Woodstock Hospital

Alexandra Hospital (Ingersoll)

Brantford General Hospital

Tillsonburg District Memorial Hospital

Alexander Marine and General Hospital (Goderich)

Children’s Hospital LHSC

Four Counties Health Services (Newbury)

Grey Bruce Health Services - Lion’s Head Hospital

Grey Bruce Health Services - Markdale Hospital

Grey Bruce Health Services - Meaford Hospital

Grey Bruce Health Services - Owen Sound Hospital

Grey Bruce Health Services - Southamptom Hospital

Grey Bruce Health Services - Warton Hospital

Hanover and District Hospital - Hanover

Listowel Memorial Hospital - Listowel

Seaforth Community Hospital - Seaforth

South Bruce Grey Health Centre - Chelsey

South Bruce Grey Health Centre - Durham

South Bruce Grey Health Centre - Kincardine

South Bruce Grey Health Centre - Walkerton

South Huron Hospital Association - Exeter

Strathroy Middlesex General Hospital

Wingham and District Hospital

Woodstock Private Hospital

Syndenham District Hospital - Wallaceburg

Cambridge Memorial Hospital

Groves Memorial Community Hospital (Fergus)

Guelph General Hospital

St. Joseph’s Heath Care System Guelph

St. Mary’s General Hospital (Kitchener)

Grand River Hospital Corporation (Kitchener) Freeport and KW Site

North Wellington Health Care Corporation (Mount Forest) Louise Marshall Hospital and Palmerston District Hospital

## Abbreviations

ASRA: American Society of Regional Anesthesia
CAS: Canadian Anesthesiologist Society
CVC: Central venous catheter
NICE: National Institute for Health and Care Excellence
POCUS: Point-Of-Care Ultrasound
UK: United Kingdom
US: Ultrasound

## Footnotes

1 Data from Wikipedia available @https://en.wikipedia.org/wiki/Southwestern_Ontario
